# Use of big data on the social determinants of TB to find the “missing millions”

**DOI:** 10.5588/ijtld.22.0080

**Published:** 2022-12-01

**Authors:** O. Biermann, T. Wingfield, B. Thapa, O. Babajide, Z. Zeinali, I. Torres, S M Abdalla, Sandro Galea

**Affiliations:** 1 Department of Global Public Health, Karolinska Institutet, Solna, Sweden; 2 Departments of International Public Health and Clinical Sciences, Liverpool School of Tropical Medicine, Liverpool, UK; 3 Department of Health Services, Policy and Practice, Brown University, Providence, RI, USA; 4 Urban Health Collaborative, Drexel University, Philadelphia, PA, USA; 5 Rockefeller Foundation, Boston University 3-D Commission on Determinants, Data, and Decision-making, Boston, MA, USA; 6 Fundación Octaedro, Quito, Ecuador; 7 Boston University School of Public Health, Boston, MA, USA

Dear Editor,

Globally, TB remains a leading cause of death by an infectious disease.^1^ TB is driven by social determinants such as poverty, food insecurity, and poor living and working conditions.^2^,^3^ Annually, 10 million people are estimated to become ill with TB, and many are missed by health services. Because of the COVID-19 pandemic, these “missing millions” have grown from 2.9 million people in 2019 to 4.2 million in 2020.^1^ The WHO and the United Nations Sustainable Development Goals (SDGs) aim to end TB disease by 2030, highlighting the need for active case-finding as part of an integrated approach to finding the “missing millions”. The data sources that are typically used to identify people with TB (e.g., survey data from demographic and health surveys) are limited in their capacity to identify areas of high risk, and therefore in their ability to have precisely targeted interventions. Understanding the social determinants of TB through the use of new data sources, including “big data” (characterised by high volume, velocity and variety), could help to reveal uncounted vulnerable populations and allow better targeted TB interventions.^4^ For example, better targeting could enhance the accuracy and yield of active case-finding strategies, help identify communities where standard TB health service delivery is of low quality, educate people about TB and reduce stigma. It could also inform efforts for TB prevention (e.g., the provision of TB preventive therapy), or the delivery of holistic support packages with broader health and socio-economic benefits.

Mining surveillance data,^5^ or poverty maps that show dimensions of poverty and food insecurity,^6^ could support the geographical tailoring of interventions to the poorest, or most food insecure populations with the highest risk of TB. Further examples of big data that could be useful are environmental data (e.g., related to economic activity, accessibility to facilities and climate change impact), and mobile phone and social media data (e.g., capturing individual and temporal aspects of people, groups and communities). As such, big data could complement and triangulate existing types of data to provide more in-depth, high-quality insights to inform decision-making (Figure). In geographical areas with limited sources of big data (e.g., a lack of smart phones), governments and other stakeholders could involve communities to provide data on social determinants (e.g., education and housing), making the data cycle complete, representative, and responsive to changing needs.

**Figure:**
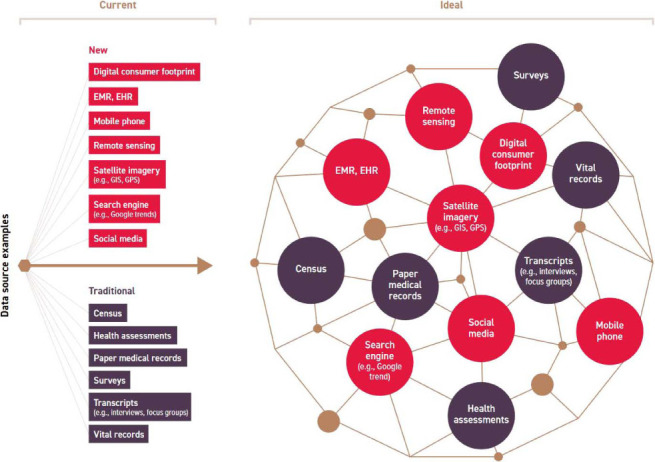
Social determinants of health data sources universe: the current state and potential for an integrated system (this figure has been developed and published by the 3-D Commission). EMR = electronic medical record; EHR = electronic health record; GIS = geographic information system; GPS = global positioning system.

Technological infrastructure continues to grow rapidly and has the potential to enhance real-time big data generation, storage and analysis. Such infrastructure includes information and communication technology with more speed, flexibility and reliability than traditional data systems (e.g., rural broadband and mobile phones).^7^ Where available, big data collected through mobile phones may help to shape the collection and use of data by communities, with substantial potential to contextualise and thus improve TB interventions. For example, self-reported data from communities has been used to inform both the local and global response during the COVID-19 pandemic, providing lessons for improvements in TB prevention and control (e.g., the *Zoe App* in the United Kingdom [covid.joinzoe.com/data] and the Johns Hopkins University [Baltimore, MD, USA] dashboard for COVID-19 [coronavirus.jhu.edu/map]), or the use of digital tools for communication and surveillance in Kerala, India.^8^ This latter example showed that digital tools were able to help meet citizens’ needs due to the high levels of collaboration and intersectoral response that connected different levels of government and multiple state departments in Kerala. These involved the private sector, and harnessed input from civil society organisations and community volunteers.^8^ In contrast to the improvements in technological infrastructure, research and data infrastructure is often lacking where this information is needed most (i.e., low-income settings with a high burden of TB). Research and data infrastructure needs to include data collection applications and approaches, and the capacity to interpret big data to address contemporary, real-world issues.^9^ However, even with infrastructure in place, disaggregated data may be unavailable, and silos of data sources often persist. This may lead to disparate databases (e.g., linked to specific goals and targets of the End TB Strategy or the SDGs), rather than a unified, collective approach. Other challenges include climate-friendly ways of storing data; legal infrastructure to regulate data privacy, security and sharing;^10^ protecting vulnerable communities, especially in authoritarian countries where data protection is problematic; and frameworks for engagement with large private and commercial firms such as Facebook/Meta (Menlo Park, CA, USA) or Google (Mountain View, CA, USA).

The use of big data to provide more in-depth, high-quality insights on the social determinants of TB, as part of an integrated approach to improve healthcare delivery, could help in the identification of the “missing millions” with TB. Despite its promise, to date, the use of big data in the field of TB has been limited to describing the global burden,^11^,^12^ predicting the flow of TB patients,^13^ optimising algorithms,^14^ and improving screening through computer-assisted diagnosis.^15^ Decision makers and international organisations (such as WHO) have an opportunity to embrace the power of big data. Dedicated international funding support could help to resolve today’s challenges related to infrastructure, research and data. Countries and institutions with strong capacity could provide secure data storage, access, and support for data analysis. In all this, safeguarding equity through equal partnerships with communities in collecting, analysing and interpreting big data (as done in the above examples for the COVID-19 response), will be critical for meaningful interventions and sustainable impact.
